# Spatial patterns and associated factors of HIV testing and counselling (HTC) as a component of antenatal care services in Ethiopia

**DOI:** 10.1371/journal.pone.0310890

**Published:** 2024-09-19

**Authors:** Tigabu Kidie Tesfie, Werkneh Melkie Tilahun

**Affiliations:** 1 Department of Epidemiology and Biostatistics, Institute of Public Health, College of Medicine and Health Sciences, University of Gondar, Gondar, Ethiopia; 2 Department of Public Health, College of Medicine and Health Sciences, Debre Markos University, Debre Markos, Ethiopia; University of Nigeria Faculty of Medical Sciences and Dentistry: University of Nigeria Faculty of Medical Sciences, NIGERIA

## Abstract

**Background:**

While HIV testing and counselling play a crucial role in preventing mother-to-child transmission, numerous pregnant women did not receive these services. Understanding the spatial variation of HIV testing and counselling and its associated factors during antenatal care in Ethiopia remains limited. Thus, this study was aimed at assessing the spatial patterns and factors associated with HIV testing and counselling during antenatal care visits in Ethiopia.

**Methods:**

A cross-sectional study design was employed with a two-stage stratified cluster sampling technique. A total of 2,789 women who gave birth in the two years prior to the survey and had at least one antenatal care visit were included in the study. Stata version 16 and ArcGIS version 10.8 software were used for analysis. A multilevel robust Poisson regression model was fitted to identify significantly associated factors since the prevalence of HIV testing and counselling was higher than 10%. A statistically significant association was declared based on multivariable multilevel robust Poisson regression analysis using an adjusted prevalence ratio with its 95% confidence interval at a p-value < 0.05. Spatial regression analysis was conducted, and the local coefficients of statistically significant spatial covariates were visualised.

**Results:**

In Ethiopia, the overall prevalence of HIV testing and counselling during antenatal care visits was 29.5% (95% CI: 27.8%, 31.2%). Significant spatial clustering was observed (Global Moran’s I = 0.138, p-value <0.001). In the spatial regression analysis, high and comprehensive knowledge related to HIV, and comprehensive knowledge on the prevention of mother-to-child transmission were significant explanatory variables for the spatial variation of HIV testing and counselling. In the multivariable multilevel robust Poisson regression analysis, education, household wealth, media exposure, number of antenatal care visits, comprehensive knowledge on mother-to-child transmission, comprehensive knowledge on prevention of mother-to-child transmission, and region were significantly associated factors.

**Conclusion:**

The prevalence of HIV testing and counselling during antenatal care visits was low. Empowering women through education, promoting mass media exposure, increasing numbers of antenatal care visits, and enhancing women’s knowledge related to HIV and mother-to-child transmission by targeting cold spot areas could improve HIV testing and counselling service uptake among pregnant women in Ethiopia.

## Introduction

Human Immunodeficiency Virus/Acquired Immunodeficiency Syndrome (HIV/AIDS) remains a major worldwide public health concern. Since the epidemic began, 84.2 million people have been infected with HIV, resulting in 40.1 million deaths from AIDS-related illnesses. In 2021, 38.4 million and 1.5 million people were living with HIV and became newly infected with HIV, respectively [1, 2]. Among people living with HIV, more than half were women [1], and 1.3 million of them were pregnant [3]. Sub-Saharan Africa carries a disproportionate burden of HIV, accounting for more than 70% of the global burden of infection and 74% of AIDS-related deaths [4].

The global aim, outlined in Sustainable Development Goal 3, is to halt the HIV epidemic by 2030 [5, 6]. The Joint United Nations Programme on HIV/AIDS (UNAIDS) introduced the 95-95-95 strategy in 2020. This strategy targets diagnosing 95% of all HIV-positive individuals, providing antiretroviral therapy (ART) for 95% of those diagnosed, and attaining viral suppression for 95% of those receiving treatment by the year 2030 [7]. Even though HIV/AIDS mortality and morbidity decreased (new HIV infections and AIDS-related deaths have been reduced by 39% and 51% from 2010 to 2023, respectively), of all people living with HIV/AIDS, only 86% knew their status worldwide in 2023 [1]. While progress towards ending the AIDS epidemic is on the right track, efforts to halt HIV transmission are lagging [6].

HIV testing and counselling (HTC) enables individuals to understand their HIV status, make informed choices, evaluate their risk of contracting HIV, and create plans to reduce their risk [8]. Timely detection of HIV during pregnancy and subsequent initiation of ART is an effective way to prevent mother-to-child transmission (PMTCT) of HIV, which is a pronounced public health problem [9]. Expanding HTC services plays a crucial role in meeting UNAIDS targets. HTC serves as a cornerstone for both preventing and treating the virus by enabling early detection. Within the 95-95-95 strategy, the initial target lays the foundation for achieving the subsequent targets. Identifying individuals as HIV-positive facilitates their prompt linkage to early HIV treatment and care. This early linkage to HIV care and treatment yields numerous benefits, including the reduction of morbidity and mortality related to opportunistic infections, decreasing mother-to-child transmission (MTCT), preventing HIV transmission to uninfected partners, enhancing quality of life, and lowering hospitalisation rates [10, 11]. Besides, through HTC, people can get accurate information on transmission mechanisms and estimate their vulnerability to the infection [12, 13]. The rationale of HTC is that if more people knew their HIV status, the number of people linked to treatment and prevention interventions would increase [14–18].

Low utilisation of HTC is a bottleneck for PMTCT, HIV treatment, and related services. Worldwide, 14% of people living with HIV were unaware of their HIV status at the end of 2022 [1]. Antenatal care (ANC) offers an opportunity for HTC, HIV services, and integrating them with routine maternal and child health services [10, 11]. Getting HTC is part of the regular ANC packages for all pregnant women [15]. Studies have identified barriers to uptake of HTC, which include poor knowledge about MTCT, low education level, fear of stigma, and poor health service access [14, 15, 19, 20].

Ethiopia is one of the sub-Saharan African countries with a high burden of HIV [21]. In 2022, the national adult HIV prevalence in Ethiopia was 0.9%, with substantial numbers of new infections [22, 23]. Ethiopia is committed to ending AIDS as a public health threat by 2030 [22]. The universal HTC for pregnant women was started in 2007 in the country [24]. However, according to a national-level meta-analysis, the pooled prevalence of MTCT in Ethiopia ranged from 7.3% to 12.6%, which is unacceptably high [25]. The status of universal HTC for pregnant women in Ethiopia is not well understood; therefore, it is difficult to know the impact of this strategy on the prevalence of HTC service uptake. This might affect the intention of policy changes and improvements. Currently, Ethiopia is committed to eliminating the epidemic by 2030 and achieving the 95-95-95 strategy targets [7]. Routine HTC is an integral aspect of HIV prevention, treatment, and care, particularly during ANC follow-up. This routine practice serves as a pivotal entry point for PMTCT of the virus [26]. In the country, almost all HTC during pregnancy has been provided as a component of ANC services, and only two-thirds of pregnant women have had ANC visits [27], which can be a barrier to improving the uptake of HTC during pregnancy. As per the national guidelines of Ethiopia, pregnant women on ANC are routinely offered HTC as part of the standard care with the right to refuse testing. The strategy follows the ‘opt-out’ approach. Pre-test counselling is provided through individual or group counselling, and clients are offered rapid HIV testing with the provision of results on the same day, and then post-test counselling is provided in consideration of the test result [28, 29]. Through the integration of HTC and routine ANC services, the accessibility and cost-effectiveness of the PMTCT services can be increased [26, 30].

Even though HTC is a universal service during ANC, a significant number of women in HIV epidemic regions haven’t been tested for HIV during their pregnancy [10, 18]. A previous study shows that HIV prevalence tends to cluster in specific geographic areas of Ethiopia [31]; this could be linked to varying utilisation of HTC services across regions. A study focusing on Ethiopian youth highlighted notable differences in HTC utilisation across different regions [13].

One study has been conducted on HIV testing among pregnant women in Ethiopia at the national level [28]. However, the clustering nature of the data wasn’t considered during the analysis. It is a fact that ignoring the effect of clustering usually results in biased and misleading conclusions [32–34]. In addition, the study only included pregnant women who gave birth one year before the survey; this resulted in the unnecessary exclusion of study subjects (reducing the sample size). According to the guide to Demographic and Health Survey (DHS) statistics, women who gave birth two years preceding the survey should be included during analysis to estimate HIV testing during pregnancy (ANC) [35]. Furthermore, this study reported the prevalence at 35.1% and quantified the association between independent factors and HIV testing using the odds ratio. Nevertheless, when the prevalence is higher than 10%, the odds ratio is not an appropriate measure of association since it misestimates the prevalence ratio. This can be resolved by using robust Poisson regression analysis [36]. Previous studies assessing the uptake of HTC during pregnancy in different locales identified health system-related and socio-demographic factors influencing HTC during pregnancy [18, 37–39]. However, these studies were conducted over a limited geographic scope, which might be difficult to generalise for all pregnant women in Ethiopia. Additionally, the spatial distribution of HTC during ANC hasn’t been explored so far. The methodological gaps [28], lack of generalizability [18, 37–39] identified from previous studies, and absence of spatial studies on HTC during ANC visits were the driving forces to conduct this study. Spatial analysis plays a crucial role in shaping health policies [40] by pinpointing regions with varying prevalence rates. Knowing one’s HIV status through HTC during pregnancy holds exceptional significance. This knowledge is crucial because women living with HIV can potentially transmit the virus to their children during pregnancy, childbirth, or breastfeeding. It is important to ensure universal access to prevention and timely HIV treatment. As far as our understanding goes, even though it is vital to design area-targeted interventions, there haven’t been nationwide studies conducted on the spatial distribution of HTC specifically among pregnant women in Ethiopia using representative data. Therefore, this study aimed to assess the spatial patterns and factors associated with HTC during ANC visits in Ethiopia using nationally representative data.

## Methods

### Study design and period

This study was conducted based on the 2016 Ethiopian Demographic and Health Survey (EDHS), a nationally representative cross-sectional study conducted from January 18 to June 27, 2016, across Ethiopia [41].

### Study setting

The study was conducted in Ethiopia, a country situated in East Africa at 9°8’42’’ North latitude and 40°29’22.8’’ East longitude [42]. Administratively, Ethiopia is divided into nine regions and two self-administrative cities [41].

### Source and study population

The source population was all women who gave birth in the two years prior to the survey and had at least one ANC follow-up in Ethiopia, while the study population was all women who gave birth in the two years prior to the survey and had at least one ANC follow-up in the selected enumeration areas.

### Sampling procedure, sample size, and data source

**Fig 1:**
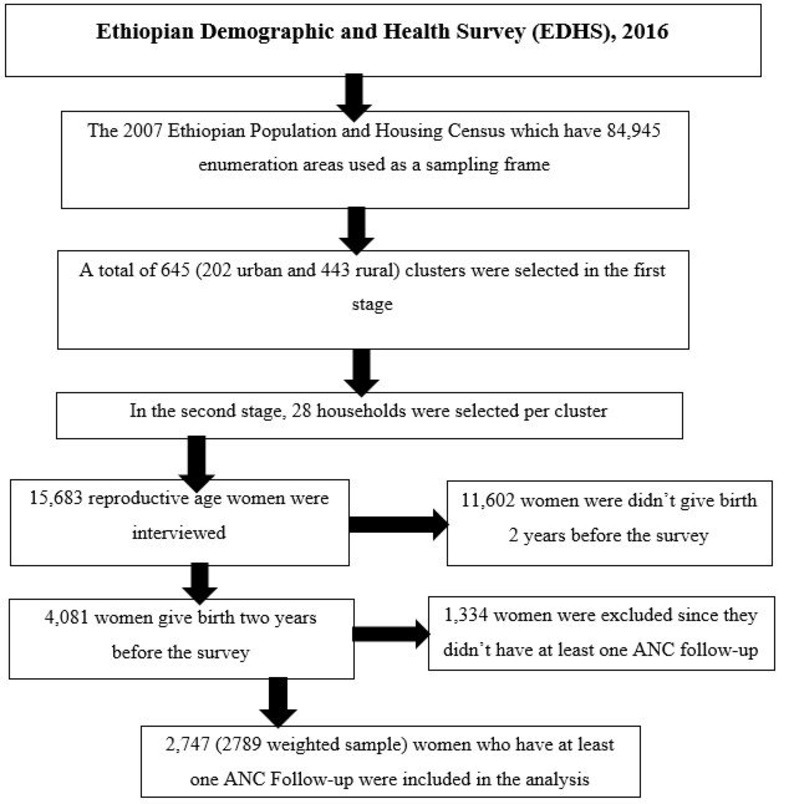
Flow diagram of EDHS 2016 sampling procedure and data extraction process.

The 2016 EDHS was conducted using a two-stage stratified cluster sampling technique. Initially, 202 urban and 443 rural (total of 645) clusters were selected based on the Ethiopian population and housing census sampling frame of 2007, which have a total enumeration area of 84,945. Next to the first stage, a fixed number of 28 households per cluster were selected randomly. A total weighted sample of 2789 women who gave birth in the two years preceding the survey and had at least one ANC follow-up was included in the study (**Fig 1**). The data used for this study was derived from https://dhsprogram.com/data/available-datasets.cfm based on an official request and permission. The authors hadn’t access to information that could identify individual participants. Additional information about data collection, sampling, and questionnaires used in the surveys was explained in the EDHS report [41].

### Variables of the study

#### Outcome variable

The dependent variable was the HTC status, categorised as either “yes” or “no.” It was classified as “yes” when all three criteria were met: (1) receiving counselling on HIV during ANC; (2) undergoing an HIV test during ANC; and (3) receiving the results of the test. Conversely, it was categorised as “no” if at least one of the following conditions was met: (1) not receiving counselling on HIV during ANC; (2) not undergoing an HIV test during ANC; or (3) not receiving the results of the test [35].

#### Independent variables

Factors associated with HTC status were selected based on studies done in different settings and populations. These include individual-level factors like age, educational status, wealth index, marital status, religion, media exposure, knowledge on where to get an HIV test, knowledge related to HIV, stigma, comprehensive knowledge related to MTCT, comprehensive knowledge related to PMTCT, risky sexual behaviour, employment, and the number of antenatal visits during pregnancy. Also, community-level factors were: residence, community knowledge related to HIV, community wealth, community women’s educational status, region, and distance from health facilities [18, 43–47].

### Measurement and operational definitions

#### HIV related knowledge

This variable was assessed the participant’s knowledge on HIV prevention and transmission mechanisms and it was created based on combination of six knowledge indicator items. These items were: (1) the risk of getting HIV can be reduced by always using a condom during sex; (2) the risk of getting HIV can be reduced by having only one sexual partner who has no other partners; (3) someone can get HIV from mosquito bites; (4) someone can get HIV by sharing food with someone who has AIDS; (5) someone can get HIV by witchcraft or supernatural means; and (6) a healthy-looking person can have HIV. Based on the correct response of the participants from the total items, it was categorised as low (score ≤ 3), high (score 4–5), and comprehensive (score 6) knowledge related to HIV [47].

#### Media exposure

This variable was derived from the participant’s engagement with media sources, including reading newspapers, listening to the radio, or watching television. It was coded as “yes” if a woman had exposure to at least one of these media channels and “no” otherwise.

#### Risky sexual behaviour

This variable was used to measure the participant’s risky sexual behavior. Five items were used to create a risky sexual behaviour index. These items were: (1) had a genital sore or ulcer in the last 12 months; (2) had a genital discharge in the last 12 months; (3) had any STI in the last 12 months; (4) had multiple lifetime sexual partners; and (5) had at least one sexual partner other than a husband in the last 12 months. These were combined into an index of risky sexual behaviour with three categories: “no risk” (score 0), “some risk” (score 1), and “high risk” (score ≥ 2) [47].

#### HIV-related stigma

To create a stigma index, six items that reflect a negative attitude towards those living with HIV were used. These items were: (1) HIV-positive children should not be allowed to attend school with children without HIV; (2) would not buy fresh vegetables from an HIV-positive vendor; (3) people with or believed to have HIV lose respect from other people; (4) people talk badly about people with or believed to have HIV; (5) people hesitate to take an HIV test due to others reactions if positive; and (6) would be ashamed if a family member had HIV. Based on the participant’s negative attitude reflected by the items, this index was then categorised as “no stigma” if the participant says no to all items (score 6), “low stigma” (score 4–5), “moderate stigma” (score 2–3), and “high stigma” (score ≤ 1) [47].

#### Comprehensive knowledge on MTCT

The women’s knowledge about the MTCT of HIV was assessed by this factor. The index was created by combining three items. These items were: (1) HIV can transmit during pregnancy; (2) HIV can transmit during delivery; and (3) HIV can transmit during breast feeding. Women were classified as knowledgeable (yes) if they were able to list at least two means of transmission (during pregnancy, delivery, or breast feeding) [18, 44].

#### Comprehensive knowledge on PMTCT

A pregnant woman is classified as knowledgeable if she knows about the availability of drugs (ART) for PMTCT during pregnancy [18, 44].

#### Community-level maternal education

It is an aggregate variable measured by the proportion of women who had a minimum primary level of education. This variable was categorised based on the median value of the proportion as low (communities with <50% of women having at least primary education) and high (communities with ≥50% of women having at least primary education).

#### Community-level knowledge related to HIV

It is an aggregated variable from the individual knowledge related to HIV as proportion of women who had high or comprehensive knowledge related to HIV. Based on median value it was categorized as low (communities with less than 50% of women had high or comprehensive knowledge) and high (communities with ≥50% of women had high or comprehensive knowledge).

#### Community-level poverty

it is an aggregated variable measured by the proportion of women with the poorest or poorer household wealth index. This variable was categorised as low (communities with <50% of women had the poorest or poorer wealth index) and high (communities with ≥50% of women had the poorest or poorer wealth index).

### Data management and analysis

Data extraction, recoding, analysis, and visualisation were done using Stata version 16, Microsoft Excel, ArcGIS version 10.8, and SaTScan version 9.6 software. To restore the representativeness of the data, weighted samples were used during the analysis. Descriptive analysis was conducted and presented in the form of texts, tables, and figures. Missing data was handled based on the DHS program guidelines [35].

#### Model building process

EDHS data has a clustering nature in which observations from the same cluster aren’t independent; the intra-class correlation coefficient (ICC) was estimated to assess the clustering effect. The ICC indicates that there was a significant clustering effect (ICC > 10%). Since the dependent variable had a binary category, a multilevel logistic regression model was expected to be applied to estimate the association between independent variables and HTC status. But, the odds ratio (OR) is a good approximation of the Prevalence Ratio (PR) when the outcome is uncommon or rare (less than 10%). When the prevalence is greater than 10%, as in our case, the PR might be misestimated by the OR, hence we employed a multilevel robust Poisson regression model to get reliable estimates [48–50].The robust Poisson regression model is used to estimate the association between independent variables and a binary outcome. A robust Poisson regression model yields results that can be interpreted as prevalence ratios, unlike logistic regression, which is interpreted in terms of OR [51].

Four models were constructed using a mixed-effect Poisson regression model with robust variance. The first model was an empty model without explanatory variables to determine the extent of cluster variation on HTC; the second model was constructed with individual-level variables; the third model was constructed with community-level variables; and the fourth model was constructed with individual- and community-level variables together. Deviance, Log-Likelihood (LL), and Akaike’s Information Criteria (AIC) were used to compare and select the best-fit model, and a model with the lowest deviance, largest LL, and smallest AIC was considered the best-fit model. Finally, the adjusted prevalence ratio (APR) with its 95% confidence interval (CI) for the best-fitted model (Model-III) was reported, and variables with p-value <0.05 in the multilevel multivariable robust Poisson regression analysis were considered significant predictors of HTC.

#### Spatial analysis

A descriptive spatial analysis was conducted to highlight the pattern of HTC distribution in Ethiopia. The global spatial autocorrelation (Global Moran’s I) statistics were used to evaluate whether the distribution of HTC during the ANC visits in Ethiopia was dispersed, clustered, or randomly distributed [52].

Once the clustering pattern is obtained, hotspot analysis is done using the Getis-OrdGi* statistics to explore areas with high and low proportions of HTC [53]. Clusters of high values could be “hot spots” (high proportion of HTC), whereas clusters of low values could be “cold spot” areas (low proportion of HTC).

The main reason for sampling is to get reliable data with less economic effort. It is difficult to collect data from all areas of the country. To predict the prevalence of HTC during ANC in un-sampled areas, Empirical Bayesian Kriging interpolation was employed [54].

The Ordinary Least Squares (OLS) and Geographic Weighted Regression (GWR) statistical models were used for exploring the spatial relationship between HTC status and the independent variables. In the spatial relationship analysis, we aimed to understand the relationship between the prevalence of HTC calculated at each cluster and the other nine (9) explanatory variables identified from the multilevel analysis based on their significance. The proportion of HTC and other independent variables was calculated for each cluster and linked to geographical data (latitude and longitude) to visualise the spatial association across places.

Ordinary Least Squares (OLS) regression modelling was performed to identify predictors of the spatial heterogeneity of HTC prevalence. OLS is a global spatial regression model for testing and explaining the relationship between the dependent and independent variables. It uses a single equation to estimate the relationship between the dependent and independent variables, with the assumption of a consistent relationship across the study area. The OLS was used as a diagnostic tool and for selecting the appropriate predictors for the Geographic Weighted Regression (GWR) model [55–57]. Multicollinearity was assessed using the Variance Inflation Factor (VIF), and no multicollinearity was observed. Model diagnostic parameters, including adjusted-R^2^, Jarque-Bera, Joint F, Joint Wald, and Koenker (BP) statistics were checked. The adjusted-R^2^ measures the amount of variation in HTC prevalence that can be explained by the explanatory variables included in the model. Its value ranges between 0 and 1, in which a model with adjusted-R^2^ values closer to 1 had better predictive performance. The Joint F and Wald statistics indicate the overall model significance, whereas the Jarque-Bera statistic indicates the presence or absence of bias in the model predictions. A significant Jarque-Bera statistic indicates nonrandom model residuals. The Koenker statistic is a measure of spatial relationship stationarity in the model. The significant p-value of Koenker statistic indicates inconsistencies in the relationship between HTC and independent variables across areas [57].

The spatial stationarity assumption failed in the OLS model; therefore, a Geographically Weighted Regression (GWR) analysis was conducted using similar dependent and explanatory variables considered in the global model. GWR is a local spatial statistical technique that assumes non-stationarity in the relationship between the dependent and explanatory variables across places [55–57]. The GWR analysis was considered after the OLS model since the Koenker statistics were significant (p-value<0.05), which means the relationships between the dependent and the independent variables change from location to location. The coefficients of the explanatory variables take different values across the study area. Mapping the GWR coefficients associated with the explanatory variables, which are produced using the GWR, provides insight for targeted interventions. The corrected Akaike’s Information Criteria (AICc) and adjusted R-squared were used for the model comparison of the OLS and GWR models. The GWR model had lower AICc and higher adjusted R-squared values, which makes it the best-fitted spatial regression model for the data.

### Ethical consideration

This study was carried out per the Demographic and Health Surveys (DHS) program relevant guidelines. After we submitted a concept paper to the DHS Program/ICF International, a letter of permission was confirmed from the Institutional Review Board of the DHS program data archivist to download the dataset. Publicly available data with no personal identifier was used, and the dataset was not shared with other bodies to maintain its confidentiality.

## Results

### Characteristics of study participants

A total weighted sample of 2,789 women who had a live birth in the two years preceding the survey and had at least one ANC visit were included in the study. Of these, 30.6% of women were aged 25–29 years, and more than half of the women (51.4%) didn’t have any formal education. The largest proportion (39.9%) of women were Orthodox Christians. More than half (58%) of women were not exposed to media, 51.5% of women had four or more ANC visits, and 99.3% of women were or had been married. Only 27.6% and 16.6% of women were working and didn’t know where to be tested, respectively. More than half (52.6%) of women had high knowledge related to HIV, 38.8% of women had moderate stigma towards people living with HIV, and 1.5% of women had high risky sexual behaviour (**Table 1**).

**Table 1:** Characteristics of study participants, EDHS 2016.

Variables	Category	Weighted frequency (n = 2789)	Percentage (%)
Age	15–19	215	7.7
	20–24	667	23.9
	25–29	854	30.6
	30–34	591	21.2
	35–39	316	11.3
	40–44	108	3.9
	45–49	38	1.4
Educational level	No education	1432	51.4
	Primary	987	35.4
	Secondary	249	8.9
	Higher	121	4.3
Household wealth status	Poorest	503	18.1
	Poorer	539	19.3
	Middle	581	20.8
	Richer	578	20.7
	Richest	588	21.1
Religion	Orthodox	1112	39.9
	Muslim	1017	36.4
	Protestant	574	20.6
	Catholic	32	1.2
	Others	54	1.9
Media exposure	No	1617	58.0
	Yes	1172	42.0
Number of ANC visits	1–3	1353	48.5
	≥4	1436	51.5
Marital status	Never	21	0.7
	Ever	2768	99.3
Employment	Not working	2020	72.4
	working	769	27.6
Know where to be tested	No	436	16.6
	Yes	2193	83.4
Knowledge related to HIV	Low	1025	36.8
	High	1468	52.6
	Comprehensive	296	10.6
Stigma	No	314	11.2
	Low	737	26.4
	Moderate	1081	38.8
	High	657	23.6
Risky sexual behavior	No	2241	80.3
	Some risk	507	18.2
	High risk	41	1.5
Knowledge on MTCT	No	792	28.4
	Yes	1997	71.6
Knowledge on PMTCT	No	1465	52.5
	Yes	1324	47.5
Residence	Urban	480	17.2
	Rural	2309	82.8
Distance to a health facility	Big problem	1487	53.3
	Not big problem	1302	46.7
Region	Tigray	291	10.4
	Afar	24	0.8
	Amhara	606	21.7
	Oromia	993	35.6
	Somali	83	3.0
	Benishangul-Gumuz	33	1.2
	SNNPR	621	22.3
	Gambela	7	0.3
	Harari	7	0.3
	Addis Ababa	108	3.9
	Dire Dawa	16	0.6
Community-level knowledge related to HIV	Low	1611	57.8
	High	1178	42.2
Community-level women’s education	Low	1578	56.6
	High	1211	43.4
Community level poverty	Low	1406	50.4
	High	1383	49.6

### Prevalence of HTC during ANC visits

**Fig 2:**
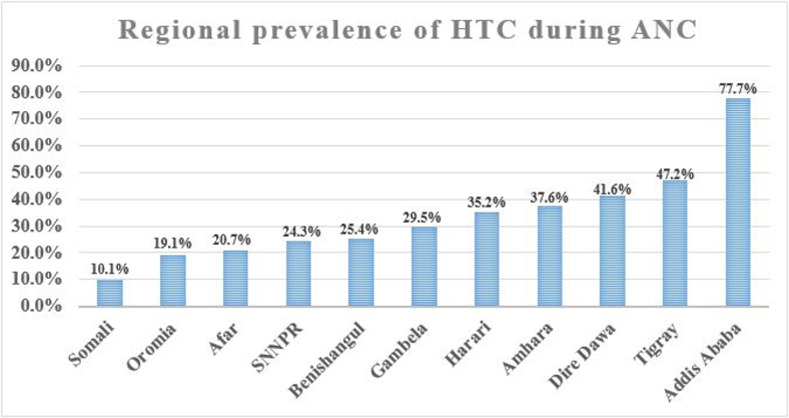
Prevalence of HTC across regions of Ethiopia, EDHS 2016.

The overall prevalence of HTC during ANC was 29.5% (95% CI: 27.8%, 31.2%). The lowest and highest prevalence of HTC were in the Somali region (10.1%) and Addis Ababa (77.7%), respectively (**Fig 2)**.

### Factors associated with HTC during ANC visits

#### Random effect analysis

The estimated ICC value of 38.37% (95% CI: 30.86%, 46.48%) indicated that about 38.37% of the overall variability in HTC as part of the ANC visit was explained by the between-cluster variation, while the remaining 61.63% was attributed to individual-level variation. Additionally, the Likelihood Ratio (LR) test (LR test mixed-effect vs. logistic model: X^2^ (01) = 151.20, p-value < 0.001) indicated that multi-level models were better fitted to the data than the classical logistic regression model. Model comparison was made among a model without explanatory variables (null-model), a model including individual-level factors (Model-I), a model including community-level factors (Model-II), and a model including individual- and community-level variables (Model-III) using model comparison parameters. The model with individual- and community-level factors together (Model-III) had the smallest AIC, the smallest deviance, and the largest Log-Likelihood (**Table 2**). Based on such information, model-III was finally used to report factors associated with HTC.

**Table 2:** Bi-variable and multivariable mixed-effect robust Poisson regression analysis to identify factors associated with HTC during ANC visits, EDHS 2016.

Variables	Category	Crude PR (95% CI)	Adjusted PR (95% CI)
Age	15–19	1.00	1.00
	20–24	0.88 (0.62, 1.25)	0.77 (0.56, 1.05)
	25–29	0.91 (0.64, 1.28)	0.78 (0.57, 1.06)
	30–34	0.94 (0.66, 1.32)	0.83 (0.60, 1.14)
	35–39	1.00 (0.69, 1.45)	0.96 (0.68, 1.34)
	40–44	0.79 (0.43, 1.43)	0.86 (0.51, 1.45)
	45–49	0.69 (0.26, 1.87)	0.85 (0.29, 2.53)
Educational level	No education	1.00	1.00
	Primary	2.28 (1.83, 2.85)^***^	1.33 (1.06, 1.65)^*^
	Secondary	2.72 (2.07, 3.59) ^***^	1.05 (0.79, 1.38)
	Higher	3.86 (2.89, 5.14) ^***^	1.20 (0.89, 1.61)
Household wealth status	Poorest	1.00	1.00
	Poorer	2.03 (1.33, 3.12) ^***^	1.60 (1.05, 2.44)^*^
	Middle	1.95 (1.16, 3.29) ^*^	1.52 (0.89, 2.58)
	Richer	3.90 (2.57, 5.93) ^***^	2.56 (1.63, 3.10)^***^
	Richest	8.34 (5.62, 12.36) ^***^	3.10 (1.90, 5.07)^***^
Religion	Orthodox	1.00	1.00
	Muslim	0.55 (0.44, 0.68) ^***^	1.15 (0.92, 1.44)
	Protestant	0.57 (0.42, 0.78) ^***^	0.94 (0.67, 1.34)
	Catholic	0.47 (0.08, 2.61)	0.93 (0.19, 4.47)
	Others	0.62 (0.18, 2.09)	1.59 (0.54, 4.71)
Media exposure	No	1.00	1.00
	Yes	3.20 (2.60, 3.94)^***^	1.37 (1.11, 1.69)^**^
Number of ANC visits	1–3	1.00	1.00
	≥4	2.08 (1.72, 2.53)^***^	1.21 (1.01, 1.47)^**^
Marital status	Never	1	1.00
	Ever	1.06 (0.44, 2.59)	1.54 (0.63, 3.80)
Employment	Not working	1	1.00
	working	1.40 (1.16, 1.68)^***^	1.09 (0.91, 1.30)
Knowledge related to HIV	Low	1.00	1.00
	High	2.26 (1.75, 2.91)^***^	1.26 (0.99, 1.60)
	Comprehensive	1.39 (0.92, 2.12)	1.24 (0.84, 1.83)
Stigma	No	1.00	1.00
	Low	1.32 (0.99, 1.76)	1.03 (0.77, 1.37)
	Moderate	1.26 (0.95, 1.68)	1.03 (0.78, 1.37)
	High	0.87 (0.61, 1.22)	1.08 (0.76, 1.53)
Risky sexual behavior	No	1.00	1.00
	Some risk	1.33 (1.09, 1.61)^**^	1.18 (0.97, 1.43)
	High risk	0.84 (0.47, 1.50)	0.66 (0.37, 1.17)
Comprehensive knowledge on MTCT	No	1.00	1.00
	Yes	3.95 (2.85, 5.47)^***^	1.64 (1.09, 2.48)^*^
Comprehensive knowledge on PMTCT	No	1.00	1.00
	Yes	4.27 (3.36, 5.42)^***^	1.90 (1.39, 2.60)^***^
Residence	Urban	1.00	1.00
	Rural	0.28 (0.23, 0.35)^***^	0.86 (0.60, 1.23)
Distance to the health facility	Big problem	1.00	1.00
	Not big problem	1.65 (1.33, 2.05)^***^	0.97 (0.80, 1.19)
Region	Tigray	1.00	1.00
	Afar	0.23 (0.10, 0.52)^***^	0.32 (0.18, 0.58)^***^
	Amhara	o.55 (0.37, 0.83)^**^	0.65 (0.46, 0.91)^*^
	Oromia	0.31 (0.18, 0.53)^***^	0.38 (0.23, 0.63)^***^
	Somali	0.16 (0.08, 0.30)^***^	0.28 (0.14, 0.56)^***^
	Benishangul-Gumuz	0.33 (0.19, 0.57)^***^	0.53 (0.33, 0.86)^**^
	SNNPR	0.48 (0.32, 0.73)^***^	0.63 (0.41, 0.95)^*^
	Gambela	0.47 (0.27, 0.80)^**^	0.49 (0.28, 0.84)^**^
	Harari	1.02 (0.68, 1.54)	0.74 (0.51, 1.05)
	Addis Ababa	1.27 (0.91, 1.78)	0.52 (0.38, 0.72)^***^
	Dire Dawa	0.97 (0.64, 1.47)	0.66 (0.47, 0.93)^*^
Community-level knowledge on HIV	Low	1.00	1.00
	High	2.71 (2.13,3.44)^***^	1.02 (0.80, 1.29)
Community-level women’s education	Low	1.00	1.00
	High	3.37 (2.59, 4.37)^***^	1.11 (0.82, 1.50)
Community-level poverty	Low	1.00	1.00
	High	0.88 (0.69, 1.12)	(0.78, 1.11)
**Comparison between models**			
**Model**	**LL**	**Deviance (-2LL)**	**AIC**
Null-model	-1154.28	2308.56	2312.57
Model-I	-1072.89	2145.89	2207.78
Model-II	-1126.72	2253.44	2287.43
Model-III	-1051.57	2103.14	2195.14

**Note**:

*P-value <0.05

**p-value <0.01

***p-value <0.001

#### Fixed effect analysis

In the fixed effect robust Poisson regression analysis variables, maternal education, wealth index, media exposure, number of ANC visits, comprehensive knowledge on MTCT, comprehensive knowledge on PMTCT, and region were significantly associated with HTC as part of ANC service.

The prevalence of HTC during ANC among women who achieved primary education was found to have increased by 33% (APR = 1.33, 95% CI: 1.06, 1.65) as compared to the prevalence of HTC among women who haven’t formal education. Women from the poorer, richer, and richest household wealth status had a 1.60 (APR = 1.60, 95% CI: 1.05, 2.44), 2.56 (APR = 2.56, 95% CI: 1.63, 3.10), and 3.10 times (APR = 3.10, 95% CI: 1.90, 5.07) higher prevalence of HTC than women from the poorest household wealth status. Being exposed to media increases the prevalence of HTC by 37% (APR = 1.37, 95% CI: 1.11, 1.69) as compared to women who hadn’t had media exposure.

The prevalence of HTC among women who had four or more ANC visits increased by 21% (APR = 1.21, 95% CI: 1.01, 1.47) as compared to women who had fewer than four ANC visits. The prevalence of HTC among women who had comprehensive knowledge on MTCT increased by 64% (APR = 1.64, 95% CI: 1.09, 2.48) as compared to the prevalence of HTC among women who didn’t have comprehensive knowledge on MTCT. The prevalence of HTC among women who had comprehensive knowledge on PMTCT increased by 90% (APR = 1.90, 95% CI: 1.39, 2.60) as compared to women who didn’t have comprehensive knowledge on PMTCT.

The prevalence of HTC was decreased by 68% among women who were living in Afar (APR = 0.32, 95% CI: 0.18, 0.58), 35% in Amhara (APR = 0.65, 95% CI: 0.46, 0.91), and 62% in Oromia (APR = 0.38, 95% CI: 0.23, 0.63), 72% in Somali (APR = 0.28, 95% CI: 0.14, 0.56), 47% in Benishangul-Gumuz (APR = 0.53, 95% CI: 0.33, 0.86), 37% in SNNPR (APR = 0.63, 95% CI: 0.41, 0.95), 51% in Gambela (APR = 0.49, 95% CI: 0.28, 0.84), 48% in Addis Ababa (APR = 0.52, 95% CI: 0.38, 0.72), and 34% in Dire Dawa (APR = 0.66, 95% CI: 0.47, 0.93) as compared to the prevalence of HTC among women who were living in Tigray (**Table 2**).

#### Spatial distribution of HTC during ANC in Ethiopia

**Fig 3:**
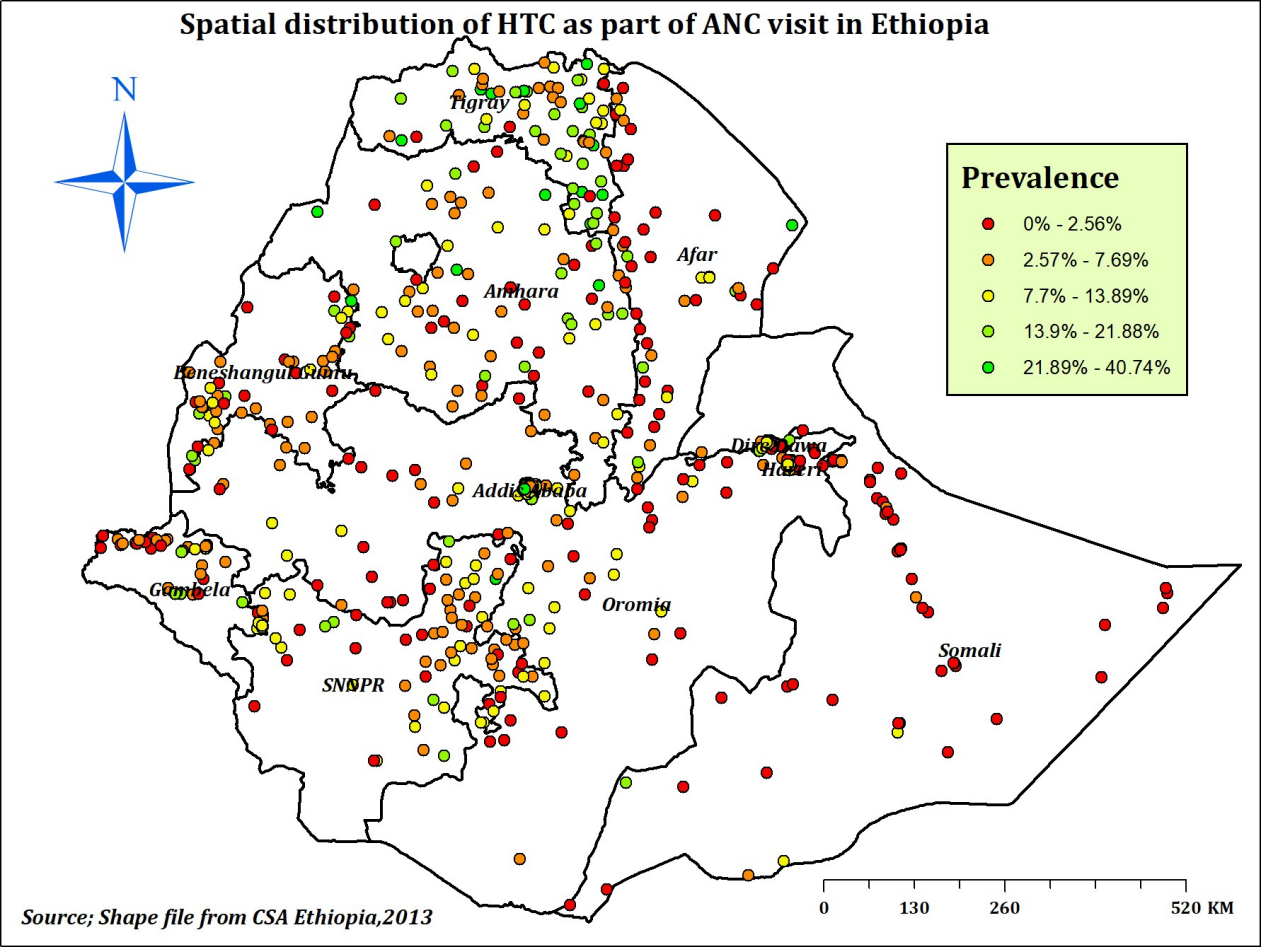
Spatial distribution of HTC during ANC in Ethiopia, EDHS 2016.

**Fig 4:**
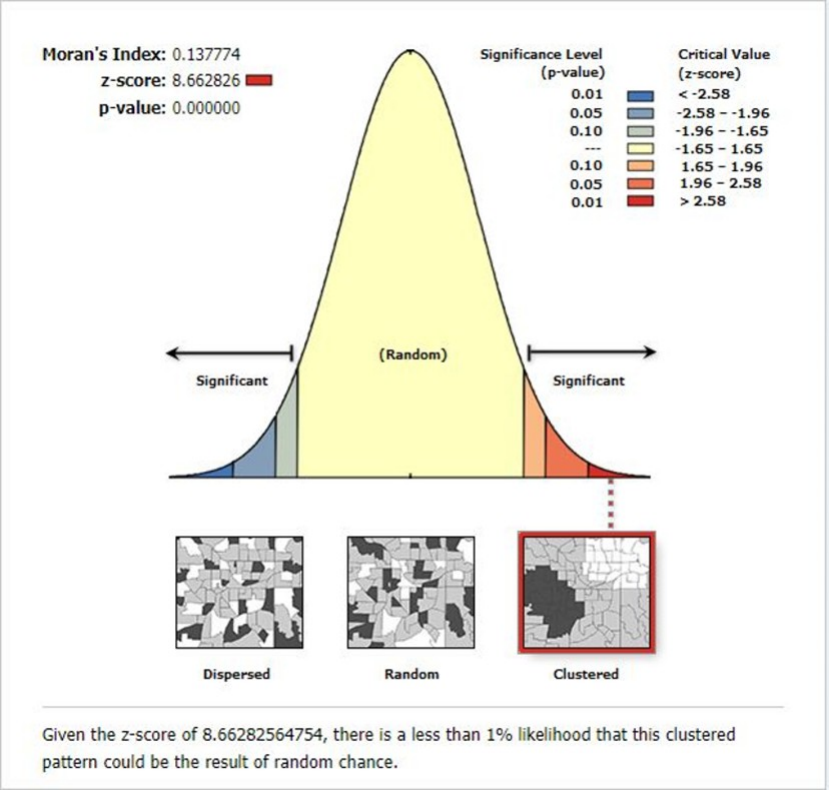
Result of the global spatial autocorrelation analysis of HTC during ANC in Ethiopia, EDHS 2016.

**Fig 5:**
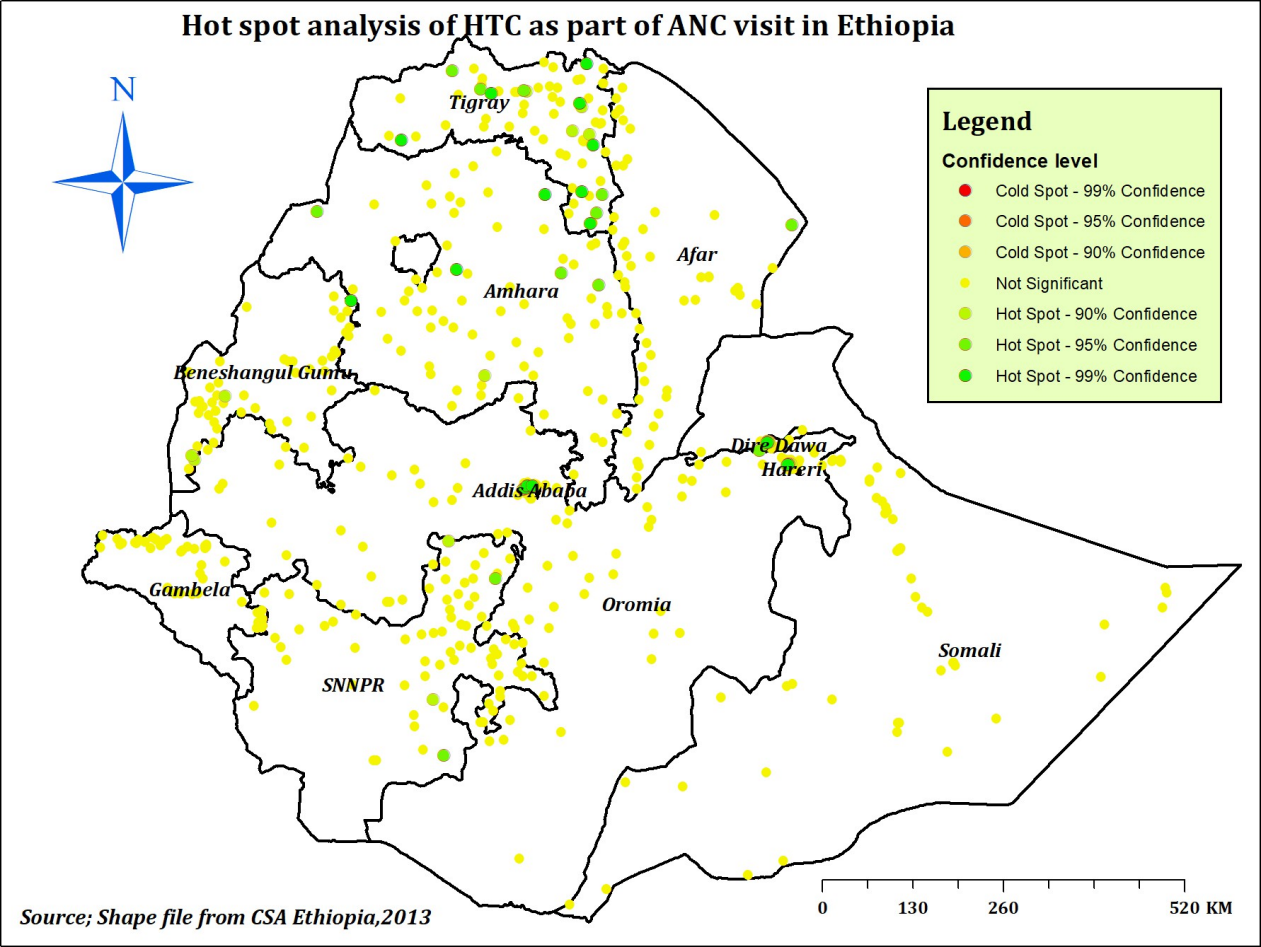
Hot spot analysis of HTC during ANC in Ethiopia, EDHS 2016.

In Ethiopia, the highest prevalence of HTC as part of ANC service was observed in northern and south-eastern Tigray, northern Amhara, Dire Dawa, and Addis Ababa (**Fig 3**). According to Global Moran’s I value = 0.138, Z-score = 8.663 and p-value < 0.001, significant spatial clustering of HTC was observed across Ethiopia (**Fig 4**). Hot spot areas (areas with a high proportion of HTC) detected with Getis-Ord GI* analysis were located in Tigray, Dire Dawa, Harari, and Addis Ababa, which are represented by green colours (**Fig 5**).

#### Spatial interpolation of HTC during ANC in Ethiopia

**Fig 6:**
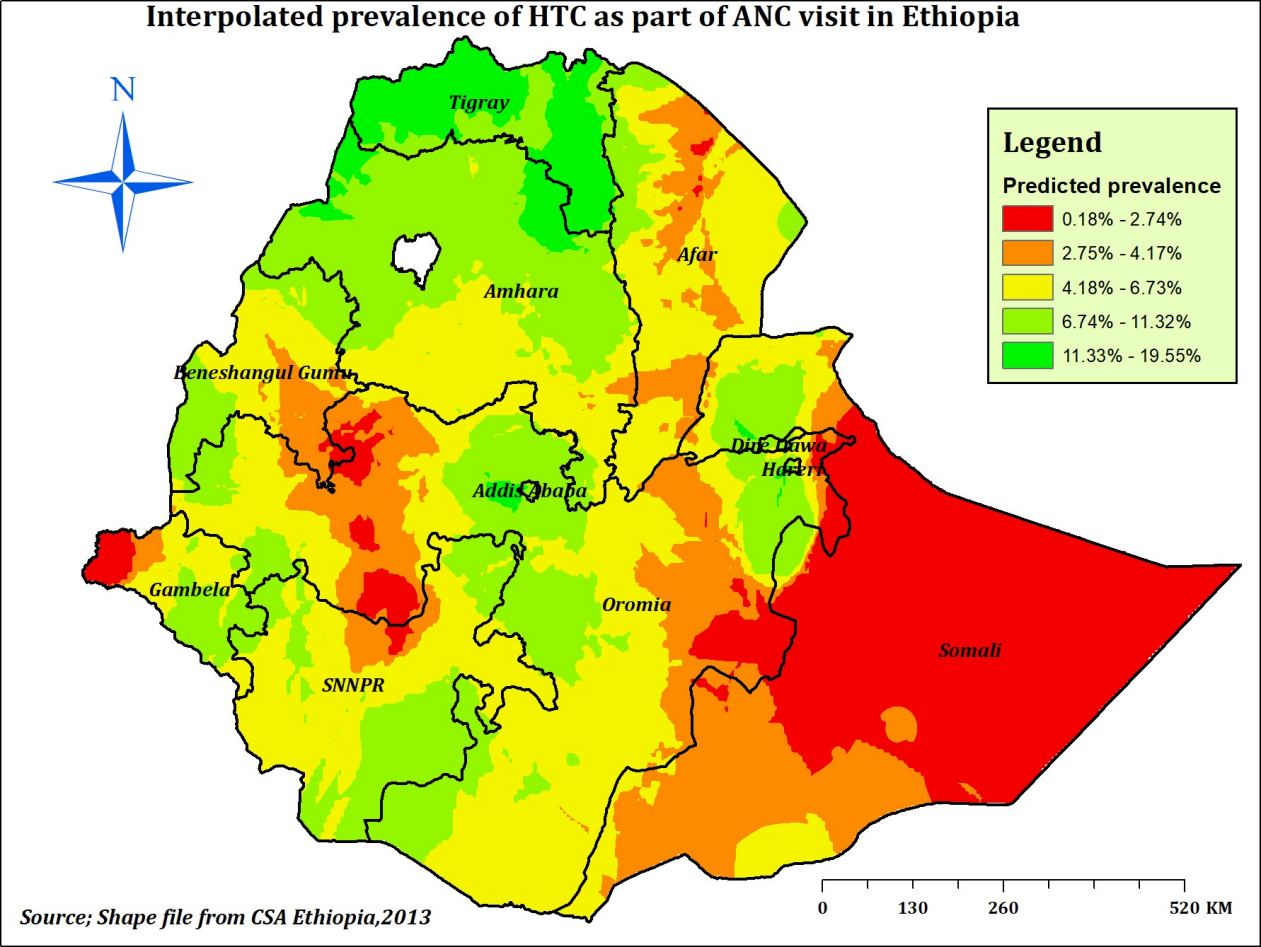
Interpolated prevalence of HTC during ANC in Ethiopia, EDHS 2016.

Empirical Bayesian Kriging (EBK) interpolation was conducted for the purpose of spatial prediction. The prediction map showed that areas represented by green colours were predicted to have a high prevalence of HTC in Ethiopia. The predicted low prevalence of HTC was also represented by red colours (**Fig 6**).

#### Global ordinary least squares regression

The OLS regression analysis was conducted to identify the spatial predictors of HTC as part of the ANC service in Ethiopia. In the OLS model, 28% of the spatial variation in HTC as part of the ANC visit was explained (adjusted-R^2^ = 0.28). Multicollinearity among independent variables was checked using the Variance Inflation factor (VIF), which was below 7.5 for each variable. The joint F-statistic and joint Wald-statistic model diagnostic parameters were significant (p-value < 0.001), which indicated the OLS model was statistically significant. According to the Jarque-Bera statistic, residuals were not normally distributed (p-value < 0.001). The Bruesch-Pagan Koenker statistic was statistically significant, which indicated the heterogeneous (non-stationary) relationship between independent variables and HTC across the study area. Based on the Koenker statistics result, the GWR model is indicated as it assumes a heterogeneous (non-stationary) relationship between the independent and dependent variables across places. The proportion of mothers who had high and comprehensive knowledge related to HIV and the proportion of mothers who had comprehensive knowledge on PMTCT were significantly associated with the proportion of HTC during ANC in the OLS regression model (**Table 3**).

**Table 3:** Ordinary least squares regression analysis results with model diagnostics and comparison parameters to identify spatial predictors of HTC, EDHS 2016.

**Variables**	**Coefficient**	**Robust standard error**		**Robust probability**
Intercept	-0.01	0.009		0.132
The proportion of mothers who had secondary and above education	-0.07	0.021		0.061
Proportion of mothers from richer and richest household	-0.02	0.013		0.052
The proportion of mothers who had exposure to media	0.02	0.016		0.162
The proportion of mothers who had ≥4 ANC visits	0.03	0.017		0.108
The proportion of mothers who had high and comprehensive knowledge related to HIV	0.06	0.016		< 0.001
The proportion of mothers who had moderate and high HIV-related stigma	0.01	0.014		0.783
The proportion of mothers who had high-risk sexual behavior	-0.01	0.060		0.975
Proportion of mothers who had comprehensive knowledge on MTCT	-0.03	0.018		0.099
Proportion of mothers who had comprehensive knowledge on PMTCT	0.13	0.024		< 0.001
**OLS model diagnostics**				
**Diagnostic parameter**	**Value**		**P-value**	
Joint F-statistic	29.12		< 0.001	
Joint Wald statistic	336.72		< 0.001	
Koenker (BP) statistic	60.96		< 0.001	
Jarque- Bera statistic	192.07		< 0.001	
**OLS and GWR model comparison parameters**				
**Model comparison parameter**	**Global model**		**GWR**	
Adjusted-R^2^	0.28		0.31	
AICc	-1749.01		-1765.35	

#### Geographically weighted regression analysis

**Fig 7:**
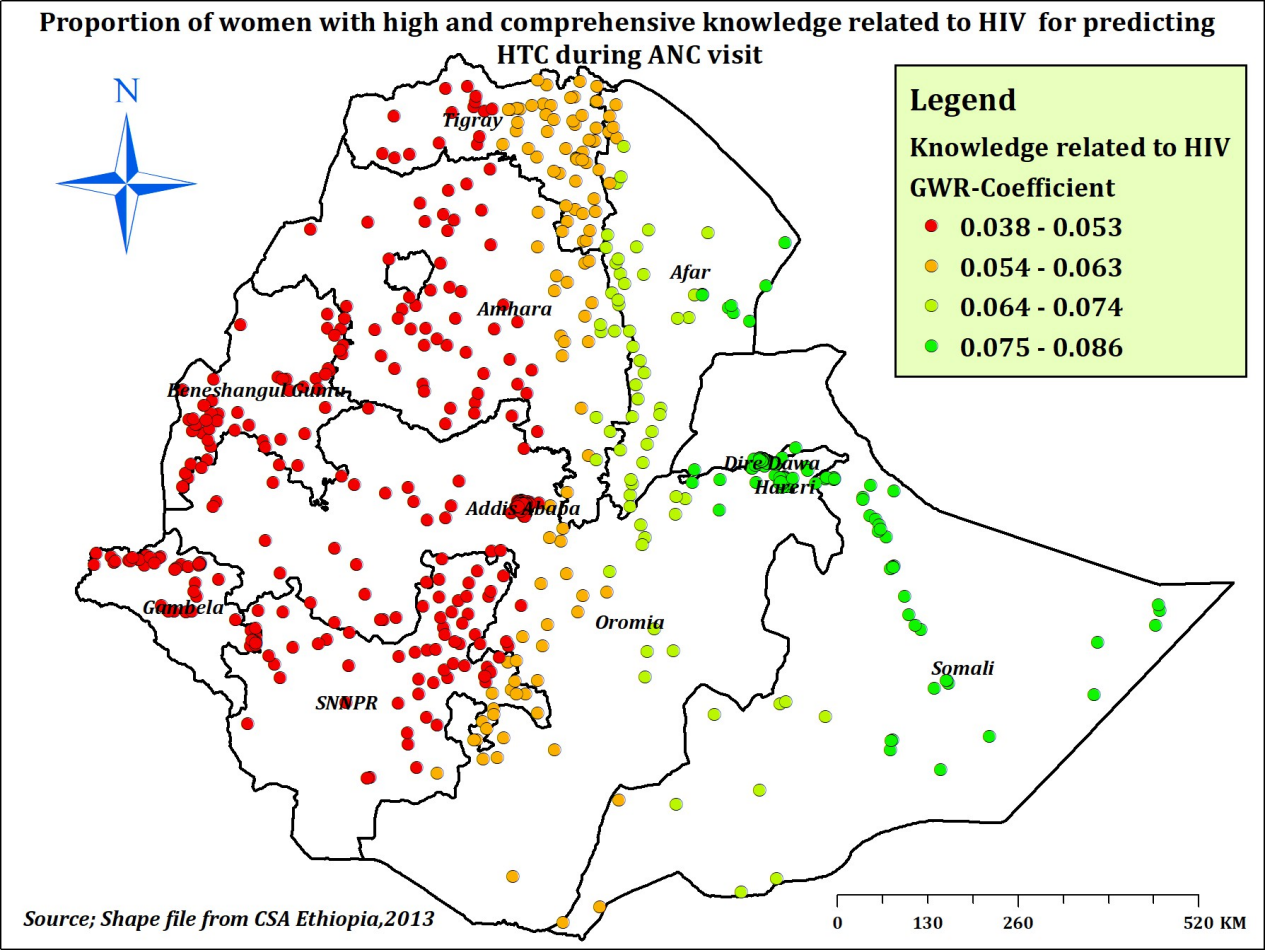
The GWR coefficients of high and comprehensive knowledge related to HIV predicting HTC prevalence during ANC in Ethiopia, EDHS 2016.

Based on the model comparison parameters, the GWR model was better than the OLS model. The ability of the models to explain variability in HTC was improved from 0.28 (OLS model) to 0.31 (GWR model). In addition, the GWR model had a significantly lower AICc value (**Table 3**). The proportion of mothers with high and comprehensive knowledge related to HIV had a significant positive relationship with the proportion of HTC. As the proportion of mothers with high and comprehensive knowledge related to HIV increased, the proportion of HTC increased in eastern Ethiopia (Afar, Dire Dawa, Harari, eastern Oromia, and Somali) (**Fig 7**).

**Fig 8:**
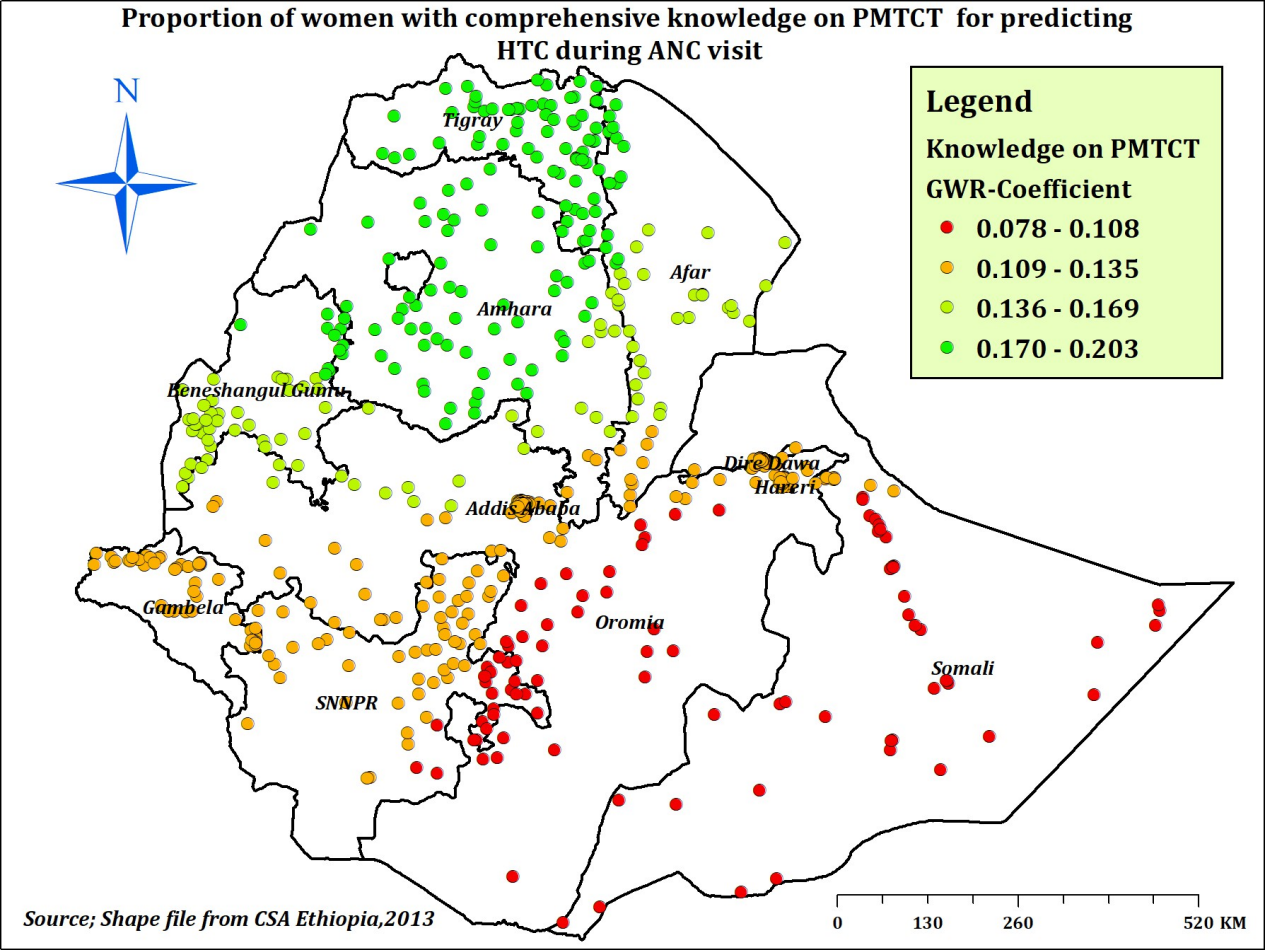
The GWR coefficients of comprehensive knowledge on PMTCT predicting HTC prevalence during ANC in Ethiopia, EDHS 2016.

The proportion of mothers with comprehensive knowledge on PMTCT had a significant positive relationship with the proportion of HTC. When the proportion of women with comprehensive knowledge on PMTCT increased, a decreased proportion of HTC was observed in Tigray, Amhara, Afar, and Benishangul-Gumuz regions (**Fig 8**).

## Discussion

Among pregnant women, HTC during ANC has been proven to reduce the risk of HIV transmission from mother to child through improving maternal knowledge about safe behaviour, ascertaining HIV status, and increasing effective antiretroviral regimen coverage [26, 30, 58]. HTC facilitates linkage to early HIV treatment, care, and support services. This yields benefits that include a reduction in morbidity and mortality related to opportunistic infections and HIV transmission to uninfected partners or children [18, 19]. In this study, factors associated with HTC were identified and spatial patterns were visualised.

In this study, the prevalence of HTC in Ethiopia as part of ANC service was 29.5% (95% CI: 27.8%, 31.2%). This is lower than the Ethiopian national ANC guideline recommendation, which indicates all pregnant women should be tested for HIV during their health facility visit as early as possible [59].

The region with the lowest prevalence of HTC was Somali, with only 10.1% utilisation. The lowest utilisation of HTC in Somali might be due to the suboptimal HIV-related knowledge and high stigma towards PLWH [60] observed in the region as compared to the majority of Ethiopia’s regions. These variations might also be explained by the wide differences in culture, education, and media exposure across regions of Ethiopia [13]. A press release by the United Nations Office for the Coordination of Humanitarian Affairs (OCHA) indicates the presence of limited HIV testing facilities, the absence of a campaign against HIV, including posters, public information, HIV testing, and counselling services, and the high level of cultural resistance regarding information that focuses on sexual behaviour in Somali [61]. The highest prevalence of HTC was observed in Addis Ababa (77.7%). This higher prevalence could be attributed to the availability of better health service utilisation, better education, and media exposure in Addis Ababa as compared to other regions. Addis Ababa is a large city in which the national campaign has become a part of everyday life, and explicit information on sexual behaviour is regularly broadcast on television. Modalities like theatres and posters usually carry messages about the importance of condom utilisation and the possible consequences of promiscuity [61].

In the multivariable mixed-effect model, education, household wealth, media exposure, number of ANC visits, comprehensive knowledge on MTCT, comprehensive knowledge on PMTCT and region were significantly associated with HTC during ANC visits.

HTC during ANC among women who had primary education increased as compared to women who didn’t have any formal education. This is in line with previous studies done in Ethiopia [13, 46] and Papua New Guinea [45]. Education confers a greater opportunity to have higher knowledge on HIV transmission and prevention and the importance of HTC services thereby it increases HTC service uptake [62].

In this analysis, household wealth status was a significant determinant of HTC, which is in line with other studies [45–47, 63]. This positive relationship might be due to women from households with higher wealth status being more likely to seek health care services and having the ability to make decisions on HTC service utilisation. Socio-economic status highly influences the knowledge of individuals regarding HIV transmission and prevention and can also impact access to health care services [64].

Exposure to mass media was significantly associated with HTC in this study, which is in line with other studies [45, 65, 66]. This might be due to the role of mass media in promoting voluntary HTC and sustaining test-seeking behaviour [67]. Information related to MTCT of HIV and PMTC of HIV can be addressed at the community level through the use of mass media, which can play a role in HTC service uptake. Mass media exposure can increase an individual’s comprehensive knowledge about HIV/AIDS; hence, it increases testing service utilisation [68, 69].

Women who had four or more ANC visits were more likely to be tested for HIV. This finding is supported by other studies done in southern Ethiopia and Malawi [26, 70]. Women with more frequent ANC visits are likely to have better information on MTCT, its prevention, and the benefit of being tested for HIV, which may increase the testing and counselling service uptake.

Women who had comprehensive knowledge on MTCT and PMTCT [26] were more likely to utilise HTC services, which is consistent with a study conducted in primary health care settings in Northern Ethiopia [18] and Adama [44]. Women who have comprehensive knowledge on MTCT and its prevention methods might understand the value of HTC for themselves and the fetus and henceforth might seek testing and counselling services.

The spatial distribution of HTC in Ethiopia was found to be non-random, and the hot spot areas were identified in Tigray, Dire Dawa, Harari, and Addis Ababa. Similarly, studies conducted in Ethiopia [13] and Nigeria [71] reported a non-random distribution of HTC across regions. The possible justification for this spatial variation of HTC during ANC across the country might be the regional disparities in socio-economic and educational status, media exposure and living conditions [13], and ANC service utilisation [72, 73]. Besides, the variation of HIV infection across the country might explain the spatial variation of HTC across Ethiopia [31]. Furthermore, the variation might be explained by the presence of variations in; the national campaign against HIV across the country, the availability of HIV testing and counselling services, and cultural resistance to information sharing about risky sexual behaviours, as indicated by OCHA [61].

In the spatial regression analysis, knowledge related to HIV and knowledge on PMTCT were significant spatial predictors of HTC. Having high and comprehensive knowledge related to HIV and comprehensive knowledge on PMTCT was found to be positively associated with HTC distribution across the country, with spatially varying associations. This might be explained by the regional variation in knowledge related to HIV in Ethiopia [60], which in turn resulted in a varying distribution of HTC. In addition, better knowledge related to HIV and PMTCT among pregnant women could enhance HTC during ANC visits since people with better HIV knowledge have less misconceptions about disease transmission and prevention mechanisms [60, 74].

### Strengths and limitations of the study

This study was done based on nationally representative data, which can improve the generalizability of the study findings. We conducted a multilevel robust Poisson regression analysis to accommodate the hierarchical nature of the DHS data and to get reliable estimates of statistical association. The DHS survey was based on the self-report of study participants, which is highly susceptible to recall bias. Women might be biased during interviews when providing information about their HTC status during ANC; this might result in social desirability bias. In addition, since this study was based on data collected through a cross-sectional design, it is difficult to show the temporal relationship between HTC and associated factors. Health professional and health facility-related factors were not included in the analysis since we used secondary data that didn’t include such characteristics.

## Conclusion and recommendations

The prevalence of HTC as part of ANC visits in Ethiopia was low as compared to the national ANC guideline recommendation indicating that universal HTC should be provided during pregnancy. Spatially, HTC had a significant variation across regions. Statistically significant spatial clustering of HTC was observed in Tigray, Dire Dawa, Harari, and Addis Ababa. Knowledge related to HIV and PMTCT were statistically significant spatial predictors of HTC prevalence. In this study, factors associated with HTC were also identified using multilevel analysis. Area-based prevention strategies targeting significant predictors of HTC are required to target the cold spots. Policymakers should design appropriate interventions like empowering women through improvement of their educational status, promoting exposure to mass media, achieving recommended ANC visit numbers, and enhancing women’s knowledge related to HIV, MTCT, and PMTCT could increase HTC service as part of ANC on cold spot areas. Researchers should investigate health facility and health professional-related factors that affect HTC during ANC in Ethiopia.

## List of abbreviations

AIC: Akake’s Information Criteria
AIDS: Acquired Immunodeficiency Syndrome
ANC: Antenatal Care
ART: Antiretroviral Therapy
CI: Confidence Interval
DHS: Demographic and Health Survey
EDHS: Ethiopian Demographic and Health Survey
EPHI: Ethiopian Public Health Institute
HIV: Human Immunodeficiency Virus
GWR: Geographic Weighted Regression
HTC: HIV testing and counselling
ICC: Intra-class Correlation Coefficient
LL: Log-Likelihood
MTCT: mother-to-child transmission
OCHA: United Nations Office for the Coordination of Humanitarian Affairs
OLS: Ordinary Least Squared
PLHIV: People Living with HIV
PMTCT: Prevention of mother-to-child transmission
PR: Prevalence Ratio
SNNPR: Southern Nation Nationality and People’s Region
UNAIDS: the Joint United Nation program on HIV/AIDS
